# IDH-1^R132H^ mutation status in diffuse glioma patients: implications for classification

**DOI:** 10.18632/oncotarget.8918

**Published:** 2016-04-22

**Authors:** Peng-fei Wang, Ning Liu, Hong-wang Song, Kun Yao, Tao Jiang, Shou-wei Li, Chang-Xiang Yan

**Affiliations:** ^1^ Department of Neurosurgery, Sanbo Brain Hospital, Capital Medical University, Beijing, China; ^2^ Department of Pathology, Sanbo Brain Hospital, Capital Medical University, Beijing, China; ^3^ Department of Neurosurgery, Beijing Tiantan Hospital, Capital Medical University, Beijing, China; ^4^ Beijing Neurosurgical Institute, Beijing, China; ^5^ Beijing Institute for Brain Disorders, Beijing, China

**Keywords:** gliomas, IDH-1^R132H^ mutation, prognosis, molecular pathology, Ki-67

## Abstract

WHO_2007_ grading of diffuse gliomas in adults is well-established. However, IDH mutations make classification of gliomas according to the WHO_2007_ edition controversial. Here, we characterized IDH-1^R132H mut^ status in a cohort of 670 adult patients with different WHO_2007_ grades of diffuse glioma. Patient characteristics, clinical data and prognoses were obtained from medical records. Patients with IDH-1^R132H mut^ were younger and had better clinical outcomes than those without mutations. Differences in age among patients with astrocytomas of different WHO_2007_ grades were eliminated after patients were grouped based on IDH-1^R132H^ status. IDH-1^R132H mut^ was present more often in patients with lower Ki-67 and MGMT protein levels and higher mutant p53 levels. Ki-67 was also strongly associated with WHO_2007_ grade independently of IDH-1^R132H mut^ status. Moreover, patients with Ki-67<30 survived longer than those with Ki-67≥30, regardless of IDH-1^R132H mut^ status. Patients in the IDH-1^R132H mut^ group with lower MGMT protein levels also had better clinical outcomes than those in other groups. Our results indicate that to better treat gliomas, IDH mutation status should be included when determining WHO_2007_ grade in glioma patients.

## INTRODUCTION

Gliomas are the most common primary brain tumors, accounting for 31% of all central nervous system tumors and 81% of malignant CNS tumors [1], and are classified into grades from I to IV on the basis of histopathological and clinical criteria established by the World Health Organization (WHO) [2]. Grade I gliomas are often circumscribed and generally curable with surgical resection alone [1, 2]. In contrast, Grade II and III gliomas are invasive and progress to higher grade lesions, with a poor prognosis [1, 2]. Glioblastomas, which are WHO grade IV gliomas, are traditionally classified as either primary or secondary if they developed from lower-grade gliomas [2]. Analyzing the specific genetic characteristics of gliomas has improved the understanding of glioma genesis and predictions of prognosis, and allows for the use of targeted treatments on an individual basis [3, 4]. IDH (Isocitrate Dehydrogenase) mutations are among the most common gene alterations in gliomas. Mutations in IDH genes occur in up to 80% of astrocytomas, oligodendrogliomas, oligoastrocytomas, and secondary glioblastomas, and in less than 10% of primary glioblastomas [5, 6], indicating that this mutation plays a key role in early gliomatogenesis [7]. Patients with grade II, III, or IV gliomas carrying IDH mutations have better overall survival [4, 8]. Additionally, accounting for IDH mutation status eliminates age differences in the prevalence of different WHO_2007_ grade gliomas [9, 10]. These results indicate that additional characteristics in addition to WHO_2007_ grade should be considered when classifying gliomas.

Studies of IDH-1 mutations are frequently based on DNA sequencing, a method which is usually considered robust. However, the IDH1 R132H antibody (clone H09) is more convenient, reliable, and consistent for the detection of IDH1^R132H^ protein, and is widely used in clinical diagnosis and research [11–15]. In this study, in addition to IDH-1^R132H mut^ protein levels, Ki-67 index and mutant P53 and MGMT (O (6)-methylguanineDNA methyltransferase) protein levels were explored in a cohort of glioma patients from a single institution in China. The predictive value of IDH-1^R132H mut^ levels for glioma patient prognosis was also investigated in this study.

## RESULTS

### IDH-1 R132H mutations in various histological types

We analyzed the distribution of IDH1^R132H mut^ in 33 astrocytomas (A), 41 oligodendrogliomas (O), and 82 oligoastrocytomas (OA). The IDH1^R132H^ mutation was present less often in A, at 60.6%, than in O or OA (87.8% and 84.1% respectively, *p*=0.006) (Table 1). We also investigated the presence of IDH1^R132H mut^ in 126 anaplastic gliomas, including 32 anaplastic astrocytomas, 39 anaplastic oligodendrogliomas, and 55 anaplastic oligoastrocytomas. The distribution of IDH-1 mutation did not differ among the anaplastic glioma subtypes (Table 1). There were 300 primary glioblastomas (pGBM), 62 secondary glioblastomas (sGBM), and 26 glioblastomas with an oligodendroglioma component (GBMO). The rate of IDH-1^R132H mut^ was 6.3% in pGBM, lower than that in sGBM (71.0%, *p*<0.001) and GBMO (46.2%, *p*<0.001). IDH-1^R132H mut^ was also more frequent in sGBM than in GBMO (*p*<0.05) (Table 1).

**Table 1:** Distribution of IDH-1 R132H mutation in different grades of glioma

WHO_2007_ grade	total	Gender (F/M)	Age (median), range	Age (mean) ± SEM	*p* value	IDH-1^R132H mut^	*p* value
**II**	156	68/88	38, 20-70	38.54 ± 0.77		125 (80.1%)	
A	33	13/20	39, 20-61	39.76 ± 1.95		20 (60.6%)	
O	41	20/21	38, 20-70	38.07 ± 1.30	0.715	36 (87.8%)	**0.006**
OA	82	35/47	37.5, 20-56	38.29 ± 1.07		69 (84.1%)	
**III**	126	59/67	44, 18-72	43.13 ± 1.09		72 (57.1%)	
AA	32	15/17	42, 18-72	42.75 ± 2.30		17 (53.1%)	
AO	39	19/20	47, 18-65	44.62 ± 1.95	0.655	25 (64.1%)	0.567
AOA	55	25/30	42, 19-69	42.31 ± 1.60		30 (54.5%)	
**IV**	388	142/246	51, 18-82	51.47 ± 0.76		75 (19.3%)	
pGBM	300	109/191	53, 18-82	51.47 ± 0.76		19 (6.3%)	
sGBM	62	23/39	44.5, 18-71	43.69 ± 1.40	**0.000**	44 (71.0%)	**0.000**
GMBO	26	10/16	47.5, 23-66	48.23 ± 2.29		12 (46.2%)	

### IDH1 R132H mutations and age

The 272 patients carrying IDH-1 ^R132H mut^ were younger than the 398 patients without the mutation (39.79 ± 0.61 vs. 50.33 ± 0.66 years, *p*<0.001) (Table 2). The association between IDH-1^R132H^ mutation and younger age in astrocytic tumors contributed to this difference (Table 2). Furthermore, we analyzed age differences in patients with astrocytic neoplasms. Patients with pGBM (51.47 ± 0.76, n=300) were older than those with AA (42.75 ± 2.30, n=32; *p*=0.001) or A (39.76 ± 1.95, n=33; *p*<0.001). Patients with AA also tended to be older than those with A, but this difference did not reach statistical significance. There were no differences in age among patients with A, AA, or pGBM in either the IDH-1^R132H-wt^ (*p=*0.347) or IDH-1^R132H-mut^ (*p*=0.062) groups.

**Table 2:** Relationship between age and IDH-1 R132H mutation in gliomas

WHO_2007_ Grade	IDH-1^R132H mut^		IDH-1^R132H wt^		*p* value
	mean ± SEM, years	median, range	mean ± SEM, years	median, range	
**Total**	39.79 ± 0.61 (n=272)	40, 20-70	50.33 ± 0.66 (n=398)	52, 18-82	**0.000**
**II**	37.58 ± 0.82 (n=125)	37, 20-70	42.45 ± 1.89 (n=31)	44, 20-61	**0.011**
A	34.05 ± 1.98 (n=20)	34, 20-55	48.54 ± 2.35 (n=13)	49, 32-61	**0.000**
O	38.22 ± 1.30 (n=36)	38, 26-70	37.00 ± 5.70 (n=5)	43, 20-49	0.844
OA	38.26 ± 1.18 (n=69)	37, 20-56	38.46 ± 2.48 (n=13)	40, 26-54	0.946
**III**	41.08 ± 1.22 (n=72)	41, 20-65	45.87 ± 1.90 (n=54)	46.50, 18-72	**0.029**
AA	37.88 ± 2.16 (n=17)	38, 20-51	48.27 ± 3.87 (n=15)	50, 18-72	**0.022**
AO	45.52 ± 2.16 (n=25)	46, 26-65	43.00 ± 3.92 (n=14)	48.5, 18-65	0.543
AOA	39.20 ± 1.80 (n=30)	39.5, 20-59	46.04 ± 2.63 (n=25)	45, 19-69	**0.032**
**IV**	42.24 ± 1.19 (n=75)	42, 22-63	51.88 ± 0.73 (n=313)	53, 18-82	**0.000**
pGBM	41.58 ± 2.57 (n=19)	40, 22-63	52.14 ± 0.78 (n=281)	54, 18-82	**0.001**
sGBM	41.77 ± 1.45 (n=44)	42, 23-61	48.39 ± 3.06 (n=18)	49, 18-71	**0.030**
GBMO	45.00 ± 3.43 (n=12)	44.5, 23-63	51.00 ± 2.99 (n=14)	51, 32-66	0.197

### IDH-1 R132H mutation and other molecular markers

As shown in Table 3, 68.0% (185/272) and 32.0% (87/272) of the IDH-1 ^R132H-mut^ and IDH-1 ^R132H-wt^ groups, respectively, had Ki-67<30. A, O, and OA patients with lower WHO grades also tended to have Ki-67<30, independent of IDH status. Mutant p53 was more prevalent in the IDH-1 ^R132H mut^ group (55.9%, 152/272) than in the IDH-1 ^R132H wt^ group (23.9%, 95/398). However, mutant p53 was only present in grade II-III astrocytomas. Decreased MGMT protein levels were observed more often in IDH-1 ^R132H-mut^ patients than in IDH-1 ^R132H-wt^ patients (76.1% vs. 58.8%, *p*<0.001). However, there was no consistent relationship between MGMT proteins level and IDH-1^R132H^ status within any of the individual glioma subtypes (Table 3).

**Table 3:** Co-occurrence of IDH-1 R132H mutation and other molecular markers

Glioma		Ki-67		*p* value	p53 mut		*p* value	MGMT		*p* value
		<30	≥30		L	H		L	H	
IDH-1^R132H mut^		185	87	**0.000**	120	152	**0.000**	207	65	**0.000**
IDH-1^R132H wt^		149	249		303	95		234	164	
IDH-1^R132H mut^	A	20	0	**0.000**	4	16	**0.017**	16	4	0.924
	AA	12	5		3	14		13	4	
	pGBM	6	13		11	8		14	5	
IDH-1^R132H wt^	A	13	0	**0.000**	9	4	**0.003**	8	5	0.718
	AA	8	7		7	8		7	8	
	pGBM	81	200		232	49		156	125	
IDH-1^R132H mut^	O	36	0	**0.000**	32	4	0.090	30	6	0.616
	AO	16	9		18	7		21	4	
	OA	69	0		27	42		58	11	
IDH-1^R132H mut^	AOA	15	15	**0.000**	9	21	0.603	21	9	0.067
	GBMO	4	8		3	9		7	5	
IDH-1^R132H wt^	OA	13	0	**0.001**	7	6	0.103	9	4	1.000
	AOA	13	12		9	16		17	8	
	GBMO	10	4		10	4		10	4	

### IDH-1 R132H mutation and prognosis

Follow-up data was available for 165 pGBM, 50 sGBM, 22 GBMO, 14 AA, 28 AO, 47 AOA, 13OA, 30 O, and 2 A patients. In multivariate analysis, we found that increased IDH-1^R132H mut^, low MGMT levels, and Ki-67<30 were associated with better prognosis.

#### IDH-1^R132H^ mutation and Ki-67<30

**Figure 1:**
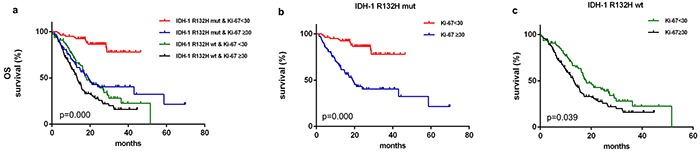
Kaplan–Meier survival curves and Breslow tests for IDH-1^R132H mut^ and Ki-67≥30 in diffuse gliomas Patient were separated into groups based on IDH-1R132H mut and Ki-67 index; clinical outcomes differed among the groups **a.** Ki-67<30 was associated with longer overall survival in both IDH-1R132H mut **b.** and IDH-1R132H wt **c.** patients.

IDH-1^R132H^ mutation was associated with better prognosis [median 58.700 months (95% CI 23.946–93.454) vs. 15.370 months in wild-type patients (95% CI 13.190–17.550); *p*<0.001, Breslow test]. The median OS of 36.230 months (95% CI 31.103–41.357) in patients with Ki-67<30 was higher than the OS of 15.370 months (95% CI 12.842–17.898) in patients with Ki-67≥30 (*p*<0.001, Kaplan-Meier method and Breslow test). We subdivided IDH-1^R132H mut^ or IDH-1^R132H wt^ gliomas based on Ki-67<30, and found differences in mOS among the four groups (Figure 1a). Patients with Ki-67<30 had better prognoses, regardless of whether they had IDH-1 ^R132H-mut^ [(median not reached) vs. 19.000 months (95% CI 13.754–24.246); *p*<0.001, Breslow test, Figure 1b] or IDH-1^R132H wt^ [median 17.400 months (95% CI 13.627–21.173) vs. 13.270 months (95% CI 11.141–15.399); *p*=0.039, Breslow test, Figure 1c]. However, OS did not differ between IDH-1^R132H mut^/Ki-67≥30 and IDH-1^R132H wt^ /Ki-67<30 patients (*p=*0.751, Breslow test).

#### IDH-1^R132H^ mutation and MGMT

**Figure 2:**
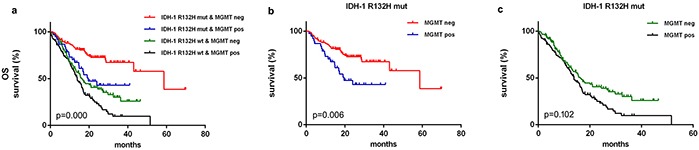
Kaplan–Meier survival curves and Breslow tests for IDH-1R132H mut and MGMT proteins in diffuse gliomas Overall survival differed depending on IDH-1R132H mut status and MGMT protein levels **a.** Lower MGMT levels were associated with better clinical outcomes in IDH-1 R132H mut **b.** but not in IDH-1R132H wt **c.** patients.

The mOS of 15.670 months (95% CI 13.273–18.067) in MGMT-positive patients was shorter than the mOS of 32.500 months (95% CI 22.439–42.561) observed in MGMT-negative patients (*p*<0.001, Breslow test). Furthermore, mOS differed among IDH-1^R132H-mut^-MGMT^neg^ (58.700 months, 95% CI 31.437–85.963), IDH-1^R132H mut^-MGMT^pos^ (19.570 months, 95% CI 11.018-28.122), IDH-1^R132H wt^-MGMT^neg^ (16.300 months, 95% CI 13.188–19.412) and IDH-1^R132H-WT^-MGMT^pos^ (13.830 months, 95% CI 11.019-16.641) patients (*p*<0.001, Breslow test, Figure 2a). MGMT^neg^ patients in the IDH-1^R132H mut^ group (*p*=0.006, Breslow test, Figure 2b), but not in the IDH-1^R132H wt^ group (*p*=0.102, Breslow test, Figure 2c), had a much better prognosis than MGMT^pos^ patients. No difference was observed in OS between IDH-1^R132H-mut^-MGMT^pos^ and IDH-1^R132H-WT^-MGMT^neg^ patients (*p*=0.471, Breslow test).

## DISCUSSION

The most frequent IDH-1 mutation type in glioma is R132H, which accounts for 88.2%-92.7% of mutations in this gene [16–18]. In an IHC study specifically detecting IDH-1^R132H mut^ using the H09 antibody, the rates were 83.0% in A II, 90.0% in O II, 100% OA II, 81.0% in A III, 88.0% in O III, 87.5% in OA III, 4% in glioblastoma, and 71.4% in sGBM [14]. The frequency of IDH-1 mutations ranged from 4-7.6% in primary glioblastoma and 73-88% in secondary glioblastoma [19, 20]. The IDH-1 R132H mutation rates detected here are similar to the ranges published previously [14, 17, 19, 21].

Also in agreement with previous findings, patients harboring IDH-1^R132H mut^ in all grades of glioma in our study were younger than those without the mutation [6, 17, 22, 23]. Moreover, IDH-1^R132H mut^ was present much more frequently in younger A, AA, AOA, pGBM, and sGBM patients. Other studies suggest a strong association between age and the prevalence of all WHO_2007_ glioma subtypes [6, 17]. In this regard, the differences between our findings and previous studies were likely due, at least in part, to small numbers of patients with particular gliomas subtypes examined here. A II IDH^mut^ and A III IDH^mut^ patients did not differ in age in a previous study [9]. While patients with A III were older than those with A II in our study, both groups were significantly younger than those with pGBM. However, there were no differences in patient age after they were separated based on IDH-1^R132H mut^. Thus, the differences in age associated with different WHO_2007_ grades may be strongly influenced by IDH-1^R132H mut^ status.

Previous studies suggested a strong correlation between IDH mutations and lower Ki-67 index, mutant p53 levels, and MGMT promoter methylation in gliomas [10, 16, 19, 24–26]. Here, we found that lower Ki-67 was associated with IDH^R132H mut^ in all WHO_2007_ glioma subtypes. However, mutant p53 expression in astrocytic tumors only differed after patients were grouped based on IDH-1^R132H^ mutation status. It is well-established that IDH mutations are associated with MGMT promoter methylation [10, 26–28]. In the present study, an association between IDH-1^R132H mut^ and MGMT protein levels was observed in glioma overall, but not within the glioma subtypes. These differences may be due to differences in the sensitivity of the detection methods [26, 29]. However, our results indicate that combining IDH-1^R132H^ status with Ki-67 index and mutant p53 and MGMT protein levels could improve prognosis predictions in glioma patients.

Recent data suggests that IDH-1 mutations, Ki-67 index, and MGMT protein levels are prognostic factors for diffuse gliomas [15, 20, 22, 24, 30–32]. Cai *et al.* found that IDH-wt plus Ki-67-low and IDH-wt plus Ki-67-high astrocytic tumor patients had different clinical outcomes. The cutoff for Ki-67 in their study was 10%, and median survival was about 2 years in Ki-67-low and 1 year Ki-67-high patients [32]. Zeng *et al.* observed that Ki-67≥30 was associated with worse prognosis in both IDH mut (median OS=566 days) and IDH wt (median OS=355 days) groups. The median OS in our study was closer to that found by Zeng *et al*. These results indicate that Ki-67 index is a reliable candidate for determining prognosis in glioma patients in addition to IDH-1 status. Although the prognostic value of MGMT protein levels is controversial [29], we found here that they were predictive of prognosis. Different IHC detection thresholds may help explain this discrepancy.

In summary, we characterized the expression of IDH-1^R132H mut^ in a large cohort of glioma patients. IDH-1^R132H mut^ was associated with specific WHO_2007_ histological grades and younger age. Age differences between different WHO_2007_ grades of astrocytoma were strongly influenced by IDH-1^R132H^ mutation status. Low Ki-67 index values occurred much more often in patients with lower WHO_2007_ grades and IDH-1^R132H^ mutation. Finally, our study indicated that Ki-67 index and MGMT protein levels, together with IDH mutation status, were predictive of prognosis in different glioma subtypes.

## MATERIALS AND METHODS

### Patients and tumor samples

Tumor samples were obtained from Sanbo Brain Hospital. Informed consent was obtained from all patients prior to the study. All experiments using human tissues were approved by the Institutional Review Board of Sanbo Brain Hospital. 670 adult patients with diffuse supratentorial gliomas were involved in the study. WHO classification of all specimens was performed by two independent neuropathologists [2]. In the case of a discrepancy, the two observers simultaneously reviewed the slides until a consensus was achieved. Clinical data, including patients’ age at diagnosis, sex, and molecular pathology, were collected. The diagnosis of GBMO (glioblastoma with an oligodendroglioma component) was made as previously described [33]. OS (overall survival) was measured from the date of operation to the death or the last known follow-up.

### Evaluation of IDH-1^R132H mut^, MGMT, mutant P53, and Ki-67 levels by immunohistochemistry

**Figure 3:**
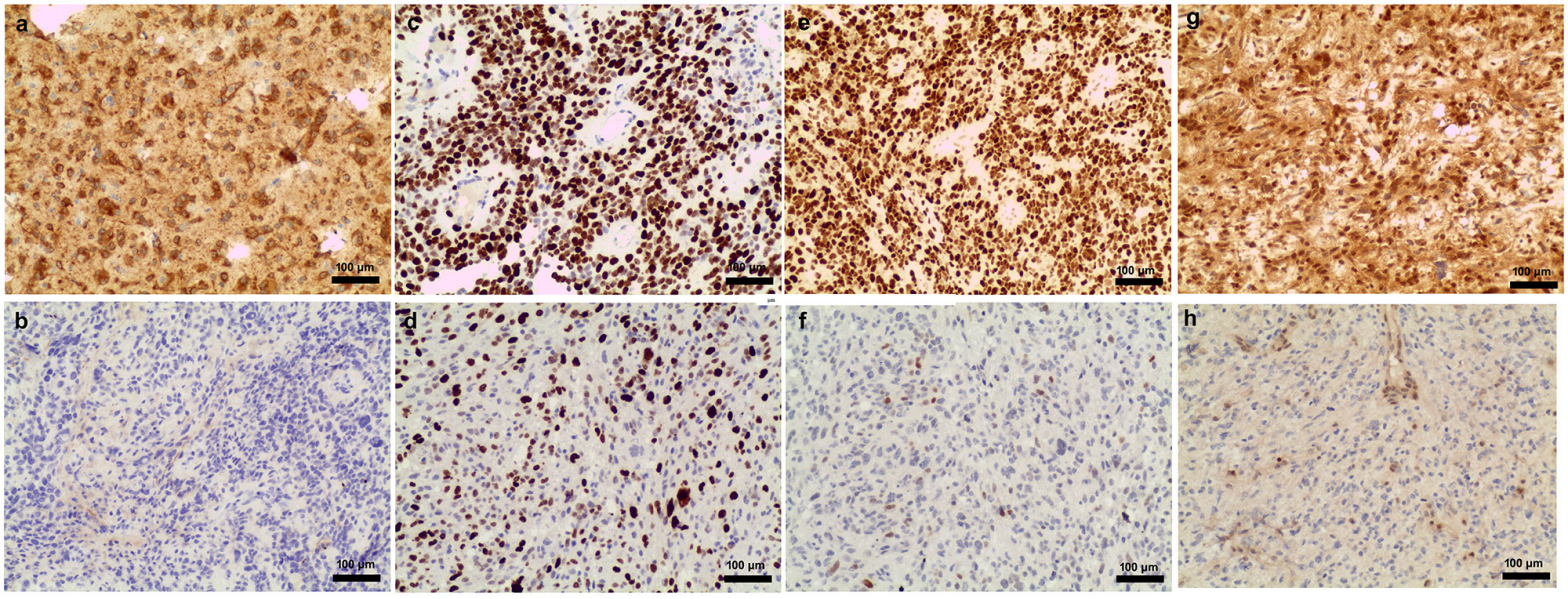
Examples of IDH-1^R132H mut^, Ki-67 index, and mutant p53 and MGMT protein levels in glioblastomas IDH-1R132H mut positive **a.** and negative **b.** Ki-67 50%-60% **c.** and 20% **d.** mutant p53 positive **e.** and negative **f.** MGMT positive **g.** and negative **f.**

Experimental procedures were performed as described previously [34–36]. Primary antibodies against IDH1^R132H^ (Dianova 1:100), p53 (1:100 Invitrogen), MGMT (1:150 Invitrogen), and Ki-67 (1:200 Invitrogen) were used. The cutoff values were 10% for IDH-1^R132H mut^, 10% for mutant p53, 10% for MGMT, and 30% for Ki-67. Representative images of high and low IDH-1^R132H mut^ (Figure 3a, 3b), Ki-67 (Figure 3c, 3d), mutant p53 (Figure 3e, 3f), and MGMT protein (Figure 3g, 3h) levels in glioblastoma patients are shown.

### Statistics

SPSS 22.0 was used for all statistical analyses. The χ^2^ test was applied to assess the co-occurrence of IDH-1 mutation patient characteristics or the presence of other disease biomarkers. Survival curves were analyzed by the Kaplan-Meier method and the Breslow test. A *p*<0.05 (two-sided) was considered statistically significant.
